# Baseline Plasma Osteopontin Protein Elevation Predicts Adverse Outcomes in Hospitalized COVID-19 Patients

**DOI:** 10.3390/v15030630

**Published:** 2023-02-25

**Authors:** Stelvio Tonello, Davide D’Onghia, Daria Apostolo, Erica Matino, Martina Costanzo, Giuseppe Francesco Casciaro, Alessandro Croce, Eleonora Rizzi, Erika Zecca, Anita Rebecca Pedrinelli, Veronica Vassia, Paolo Ravanini, Maria Grazia Crobu, Manuela Rizzi, Raffaella Landi, Luigi Mario Castello, Rosalba Minisini, Gian Carlo Avanzi, Mario Pirisi, Daniele Lilleri, Mattia Bellan, Donato Colangelo, Pier Paolo Sainaghi

**Affiliations:** 1 Department of Translational Medicine, Università del Piemonte Orientale (UPO), 28100 Novara, Italy; 2 CAAD, Center for Autoimmune and Allergic Diseases, Università del Piemonte Orientale (UPO), 28100 Novara, Italy; 3 COVID-19 Unit, Department of Internal Medicine, AOU “Maggiore della Carità”, 28100 Novara, Italy; 4 COVID-19 Sub-Intensive Unit, Division of Emergency Medicine, AOU “Maggiore della Carità”, 28100 Novara, Italy; 5 Unit of Microbiology and Virology, Department of Laboratory Medicine, Maggiore della Carità Hospital, 28100 Novara, Italy; 6 Division of Internal Medicine, Azienda Ospedaliera “SS. Antonio e Biagio e Cesare Arrigo”, 15100 Alessandria, Italy; 7 Unit of Microbiology and Virology, IRCCS Policlinico San Matteo Foundation, 27100 Pavia, Italy; 8 Rheumatology Unit, AOU “Maggiore della Carità”, 28100 Novara, Italy; 9 Department of Health Sciences, Pharmacology, Università del Piemonte Orientale (UPO), 28100 Novara, Italy

**Keywords:** Osteopontin (OPN), COVID-19, SARS-CoV-2, inflammation, biomarker

## Abstract

More than three years have passed since the first case, and COVID-19 is still a health concern, with several open issues such as the lack of reliable predictors of a patient’s outcome. Osteopontin (OPN) is involved in inflammatory response to infection and in thrombosis driven by chronic inflammation, thus being a potential biomarker for COVID-19. The aim of the study was to evaluate OPN for predicting negative (death or need of ICU admission) or positive (discharge and/or clinical resolution within the first 14 days of hospitalization) outcome. We enrolled 133 hospitalized, moderate-to-severe COVID-19 patients in a prospective observational study between January and May 2021. Circulating OPN levels were measured by ELISA at admission and at day 7. The results showed a significant correlation between higher plasma concentrations of OPN at hospital admission and a worsening clinical condition. At multivariate analysis, after correction for demographic (age and gender) and variables of disease severity (NEWS2 and PiO2/FiO2), OPN measured at baseline predicted an adverse prognosis with an odds ratio of 1.01 (C.I. 1.0–1.01). At ROC curve analysis, baseline OPN levels higher than 437 ng/mL predicted a severe disease evolution with 53% sensitivity and 83% specificity (area under the curve 0.649, *p* = 0.011, likelihood ratio of 1.76, (95% confidence interval (CI): 1.35–2.28)). Our data show that OPN levels determined at the admission to hospital wards might represent a promising biomarker for early stratification of patients’ COVID-19 severity. Taken together, these results highlight the involvement of OPN in COVID-19 evolution, especially in dysregulated immune response conditions, and the possible use of OPN measurements as a prognostic tool in COVID-19.

## 1. Introduction

The glyco-phosphoprotein osteopontin (OPN) is a constitutive part of the extracellular matrix of different tissues such as bone, kidney, and epithelial cells and is involved in several functions such as wound healing, bone turnover, tumorigenesis, and ischemia and, in the soluble form, is a regulator of inflammatory response [1,2]. OPN receptors are integrins and CD44 variants that promote adhesion, migration, and survival in different cell types [3]. This cytokine is encoded by a gene in a cluster of “SIBLING” family proteins (Small Integrin Binding Ligand N-linked Glycoprotein) located on chromosome 4 (4q13) [1,3]. Highly conserved sequence motifs, together with post- translational modifications, contribute to the multifunctional nature of OPN [4]. Although OPN is classified as a pro-inflammatory cytokine [5], it has also shown a role in inflammation, including in COVID-19 [6].

OPN’s role in innate immunity is demonstrated by its protective function in infectious diseases [7]. In fact, it was shown to contribute to the mucosal defense against viral pathogens [8]. Low concentrations of OPN are usually detected in healthy subjects, while high OPN plasma levels have been associated with chronic inflammation states and in the pathogenesis of autoimmune diseases, such as systemic lupus erythematosus, rheumatoid arthritis, and cancer [9,10,11,12]. In particular, OPN is involved in the inflammatory response during the infection of pathogens such as bacteria and viruses by recruiting neutrophils and macrophages at site of infection, activating T cells, and triggering the cytokine response [2,13,14]. SARS-CoV-2 (severe acute respiratory syndrome coronavirus 2) is a positive single-stranded RNA virus responsible for the COVID-19 pandemic [15] responsible for more than 6 million deaths worldwide [16]. SARS-CoV-2 infection can lead to a variety of clinical manifestations ranging from an asymptomatic or mild flu-like syndrome to severe cases with interstitial pneumonia with respiratory failure, requiring assisted ventilation, that may evolve into multi-organ failure and eventually death; it is often difficult to predict at hospital admission [7,17,18,19]. In most severe cases, in fact, SARS-CoV-2 infection determines an acute, uncontrolled inflammatory reaction (cytokine storm) in the lungs [20]. In this context, some authors have highlighted the role of matrix metalloproteinases (MMPs) such as MMP-8 and MMP-2 in upregulating the immune response and the activation of inflammation mediators including OPN. Moreover, high MMPs levels in plasma have been associated with the damage of lung parenchyma [21]. Additionally, it has been shown that there is a loss of interferon (IFN) production after SARS-CoV-2 infection at the pulmonary level. Consequently, there is a rapid activation of pathogenic Th1 cells that secrete pro-inflammatory cytokines such as interleukins (IL-1β, IL-6, IL-8, and IL-12), interferon-γ-inducible protein 10 (IP-10), monocyte chemoattractant protein 1 (MCP-1), and interferon-γ (IFN-γ) [3,7]. In this inflammatory contest, there are complex activities mediated by multiple cells and factors that influence the immune system and epithelia activities. The rationale of our study was to assess of OPN involvement in this framework since it is known to play either a physiologic or pathophysiologic role [1,2,3,8]. The present study evaluates the role of circulating OPN levels as a potential biomarker of COVID-19 severity and as a prognostic tool for clinical practice.

## 2. Patients and Methods

### 2.1. Patients

We conducted a prospective observational study that included 133 COVID-19 hospitalized patients in COVID-19 wards (including sub-intensive units) of the “AOU Maggiore della Carità” in Novara. These patients were enrolled between January and May 2021. We included in the study only patients that gave a signed informed consent. The inclusion criteria were age > 18 years, assessment of SARS-CoV-2 positivity by antigenic test or RT- PCR, with disease symptoms that did not exceed 12 days. The exclusion criteria were advanced oncological disease, advanced renal failure (stage V), and clinical conditions suggesting irreversibility or that led to immediate ICU admission. After signing the informed consent, patients underwent venous blood sampling upon entry and after 7, 14, and 21 days when possible. Patients were treated, unless contraindicated, following the standard COVID-19 care protocol imposed by the “AOU Maggiore della Carità” (oxygen mask, glucocorticosteroids, and low-molecular-weight heparin (LMWH)). These patients are part of a larger multicentric observational study cohort called the BIAS study (Baseline Immunity status effect on SARS-CoV-2 presentation and evolution: comparison between immuno-competent and immunocompromised patient study). The study protocol was approved on 14 January 2021 by the local Ethical Committee (CE 7/21) and was conducted in strict accordance with the Declaration of Helsinki.

#### 2.1.1. SARS-CoV-2 Variants

**Figure 1:**
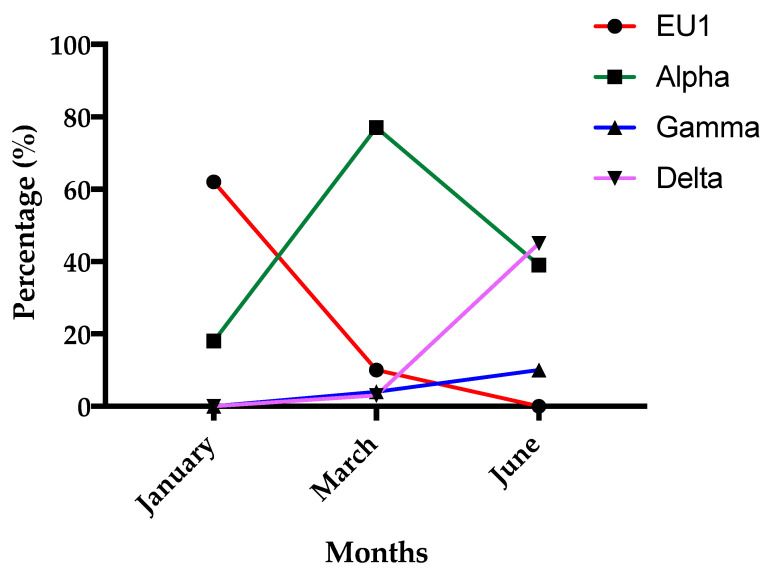
The graph shows the trend of the prevalence of SARS-CoV-2 variants in Italy in the period between 26 March 2021 and 30 June 2021.

In the period from 26 March 2021 to 30 June 2021, the Microbiology and Virology Laboratory of the “AOU Maggiore della Carità” in Novara carried out 236 analyses for the detection of SARS-CoV-2 variants by using a multiplex RT-PCR Real-Time assay for the simultaneous detection and identification of the presence of mutations in the S gene of the SARS-CoV-2 virus RNA, which are responsible for the Alpha variants of the virus (B.1.1.7 lineage) and the Beta (B.1.351 lineage), and Gamma (P 1 lineage) variants of the virus (Seegene MuDT™, Seegene Inc., Arrow Diagnostics, Italy). Data revealed a prevalence of Alpha V1 variant characterized by the presence of S N501I gene mutation and 69/70 deletion (141 of 236 cases) and a low presence of Beta V2 and Gamma V3 variants (48 of 236 cases) characterized by the presence of S N501I gene mutation and E484K deletion. The trend of the prevalence of the variants in the first semester of 2021 in Italy is shown in Figure 1. In 47 cases, it was not possible to determine the variant. No cases of Omicron were reported in our cohort and in general in Italy in the first 6 months of 2021. These results were in accordance with the overall incidence in Italy in that period. Furthermore, this is in accordance with WHO data that demonstrated that Omicron appeared in Italy starting from December 2021. Although we had no complete variant characterization for all the patients included in this study, we assumed a similar proportion incidence of variants.

#### 2.1.2. Circulating OPN Levels Determination

Plasma OPN was measured by enzyme-linked immunosorbent assay (ELISA) using a kit commercially available (R&D Systems DuoSet Elisa DY6488, McKinley, MN, USA). A Victor X4 microplate reader (Perkin Elmer, Waltham, MA, USA) was used to measure the absorbance values. A calibration curve in a range of 0–1000 pg/mL range was used for sample amount determination, as suggested by the manufacturer.

#### 2.1.3. Endpoint Definition

The correlation between OPN plasma levels at admission and at day 7 of hospitalization and the disease progression represented the endpoint. The progression was defined as unfavorable (death or admission in ICU) or recovery (National Early Warning Score 2 (NEWS2) ≤ 2 for at least 24 h in the first 2 weeks or discharge).

#### 2.1.4. Blood Sample

Blood samples were obtained in EDTA vacutainer at two time points, namely at hospital admission (baseline, t0) and at day 7 (t7), and immediately processed. Samples were stored at −80 °C.

#### 2.1.5. Routine Laboratory Evaluation

Blood samples from each patient were analyzed in clinical practice to obtain a complete cell count; a routine biochemistry panel including creatinine, alanine aminotransferase (ALT), and aspartate aminotransferase (AST); and an inflammatory panel composed of coagulation/fibrinolysis (D-dimer), ferritin, and C-reactive protein (CRP).

#### 2.1.6. Data Collection

We stored in a web-based encrypted database (REDCap platform) patients’ laboratory parameters, clinical parameters, demographics, and therapeutic schedule. We reviewed medical records for each patient and collected relevant clinical data from hospital admission (t0, baseline) to study exit (either positive or negative diagnosis, up to a maximum of 28 days).

#### 2.1.7. Statistical Analyses

Clinical and laboratory data were obtained from REDCap database and correlated with OPN levels to evaluate if there was a statistically significant correlation toward the required endpoint. The continuous variables were expressed as medians and interquartile range (IQR) to describe central tendency and dispersion. The frequencies (percentages) were used to describe the categorical variables. Mann–Whitney U-test was used to perform the statistical analyses for continuous variables. The multivariable regression models were built with the statistically significant values, as identified by univariate analysis. We built receiver operator characteristics curves (ROC) for the parameters of interest to determine the prognostic cut-off. The statistically significant threshold was set at *p* < 0.05 (two-tailed). Graphs were created using GraphPad Prism 9.4.0 (GraphPad Software, La Jolla, CA, USA). Statistical analyses were performed with MedCalc^®^ Statistical Software version 20.014 (MedCalc Software Ltd., Ostend, Belgium) and Statistica for Windows release 12 (TIBCO Soft-ware Inc, Palo Alto, CA, USA.

## 3. Results

**Figure 2:**
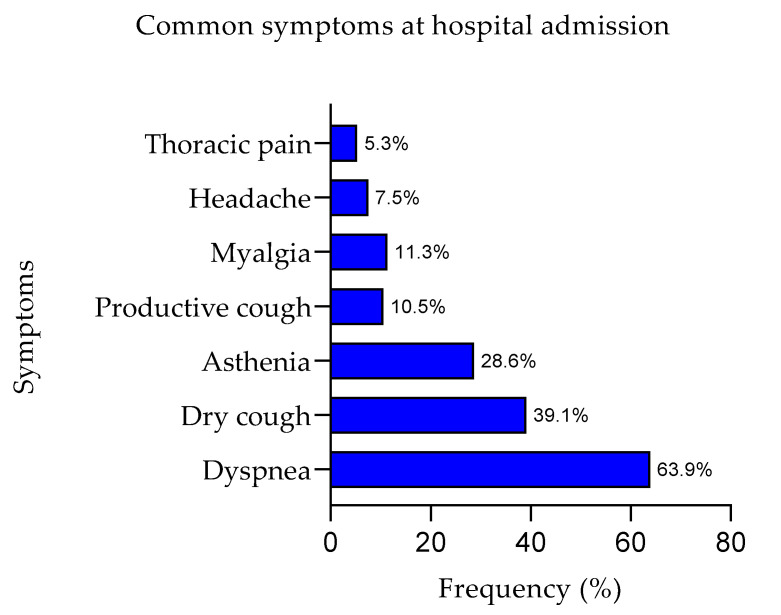
The graph shows the prevalence of symptoms at hospital admission expressed as percentage.

During the Italian third pandemic wave, from January to May 2021, 133 patients admitted to non-ICU wards of “Maggiore della Carità” University Hospital (Novara, Italy) for moderate or severe COVID-19 were enrolled and followed-up prospectively. Table 1 and Figure 2 summarize detailed demographic and hospital admission (baseline, t0) clinical conditions of the patient cohort included in this study.

Overall, 74.4% of the enrolled patients at hospital admission day showed moderate respiratory failure (100 ≤ PiO2/FiO2 < 200), while 6.8% had a severe clinical presentation (PiO2/FiO2 < 100). Some patients were already receiving COVID-19-related treatment before hospital admission. Main treatments included corticosteroids (53.4%), azithromycin (35.3%), and heparin (30.8%). The median baseline NEWS2 confirmed the severity of clinical presentations with a score of 5, IQR 4–6 [15]. In this 133-patient cohort, 29 patients (21.8%) had an outcome that was negative since there was ICU admission or death, while 87 patients (64.44%) reached a National Early Warning Score 2 (NEWS2) ≤ 2 for at least 24 h in the first 2 weeks or discharge. Interestingly, OPN levels at hospitalization day correlated with clinical evolution of the patients, as shown in Table 2. In fact, patients with significantly higher OPN levels were associated with negative progression of the disease. Furthermore, our data showed that patients with lower OPN plasma concentration at hospitalization day had faster clinical recovery ((NEWS2) ≤ 2 for at least 24 h in the first 2 weeks or discharge) although this difference was not statistically significant, as shown in Table 3. Moreover, the correlation between OPN levels and clinical output lost its significance at day 7 from hospitalization, as described in Table 2 and Table 3.

Multivariate analysis demonstrated the prognostic role of OPN towards a negative end point and disease severity parameters at admission, such as NEWS2 and PiO2/FiO2, was retained after the correction for demographic variables such as gender and age, as shown in Table 4.

Moreover, we analyzed any possible correlation between OPN levels at baseline (t0) and other laboratory parameters related to clinical severity or inflammation status. Table 5 shows the significant correlation with C-reactive protein (CRP), IP10, and MCP-1.

**Figure 3:**
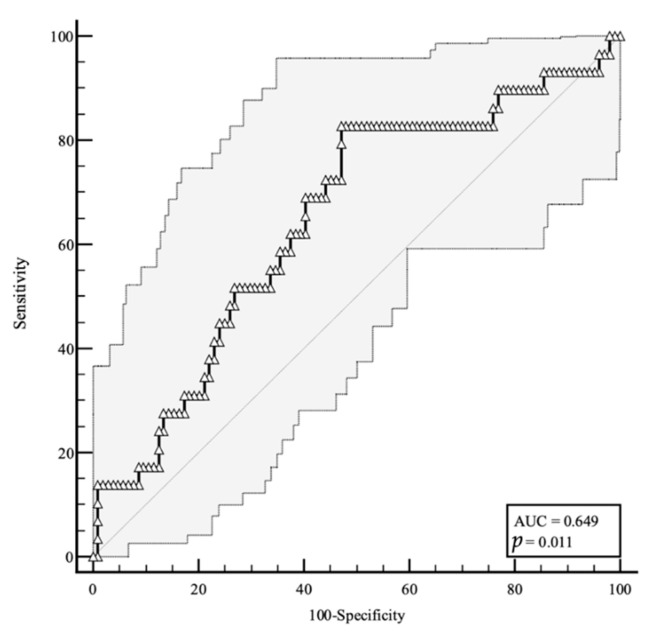
This graphical plot illustrates ROC curve for plasma OPN levels at the time of hospital admission predicting a severe disease evolution. As shown in figure, higher OPN values are predictive of a negative disease evolution. AUC, area under the curve; *p*, *p*-value.

We built an ROC curve for baseline plasma OPN to predict the adverse prognosis based on the results obtained from the correlation analyses. For the ROC analyses, sensitivity, defined as “positivity in disease”, refers to the proportion of subjects who have the target condition and are considered true positives, while specificity is defined as “negativity in health” and refers to the proportion of subjects without the target condition that are true negatives. In our simulation, we defined one target condition: the severe disease evolution. Figure 3 shows that OPN levels higher than 437 ng/mL are predictive of a negative disease evolution (area under the curve (AUC) = 0.649, 83% sensitivity, 53% specificity), with a likelihood ratio of 1.76 (95% confidence interval (CI): 1.35–2.28).

## 4. Discussion

In this prospective observational study, we evaluated the plasma concentration of OPN in a cohort of 133 hospitalized COVID-19 patients at the “AOU Maggiore della Carità” in Novara (Italy), enrolled between January and May 2021.

The Microbiology and Virology Laboratory of the hospital “AOU Maggiore della Carità” in Novara routinely performed variant analyses in infected patients. During the period considered in our study, from January 2021 to May 2021, clinical data showed that the prevalent variant in our patients was Alpha (B.1.1.7 lineage), characterized by the presence of the S N501I gene mutation and the 69/70 deletion. During the months in which patients were enrolled, other variants were also found, including the Beta V2 (B.1.351 lineage) and Gamma V3 (P 1 lineage), characterized by the presence of the S N501I gene mutation and the E484K deletion. In the last months in which we enrolled patients, the variant that infected most patients were Delta. No Omicron variant was detected during this period, in accordance with national survey that reported that Omicron appeared in Italy in December 2021. In our cohort, the variant characterization was determined for most patients, and although for some patients, it was not available since at the time of recruitment the patients could be admitted with an antigenic test, it reflected the overall incidence in this period. The virulence and the experimental data here described are thus related to the EU1, Alpha, and Gamma but not Omicron variant.

In fact, the Alpha variant appeared in December 2020 and in early February 2021 accounted for approximately 25% of cases and reached its peak in mid-April, accounting for more than 90% of cases. The Beta variant was reported in few patients. In Italy and in our region, the Gamma variant appeared at the beginning of January and peaked at in the first week of May, with around 15% of cases. The Delta variant appeared in Italy in March but with very few cases (less than 3%) and increased considerably at the beginning of June, peaking between July and November 2021. In general, in the beginning of January 2021, in Italy, we 62% of cases were EU1 and 18% Alpha and in mid-March, 77% were Alpha, 10% EU1, and 4% were of the Gamma variant. At the end of June, 39% were Alpha, 10% Gamma, and 45% Delta variant.

We observed that baseline plasma OPN levels in these patients directly correlated with clinical status, and higher OPN levels were demonstrated in patients with adverse prognosis and disease progression with respect to all other patients.

The principal pathogenetic mechanism associated with disease severity and death in COVID-19 patients is an excessive inflammatory response to SARS-CoV-2 infection, which is associated with vascular damages [22,23,24,25]. In this context, OPN might have a pivotal role since it is known to be involved in thrombosis pathophysiology driven by chronic inflammation [26,27]. Indeed, the activation of the innate immunity is responsible for severe hyperinflammatory status, and high levels of OPN correlate to a hyper activation of the immune system that might result in cytokine storm [28,29,30]. In COVID-19, there is a lung-damaging process that involves multiple factors and direct or undirect activation of hyper inflammation due to specific actors such as, for example, MMPs [21]. Since alveolar impairment in this disease is due to vessel injury and local diffuse thrombotic damage [31], high circulating levels of OPN might be associated with the initiation or progression of these events, along with a beneficial early-response mechanism to limit viral infection damages [32]. Noticeably, we also demonstrated a significant correlation between OPN and high levels of C-reactive protein, MCP1, and IP10.

These proteins are known to be involved in initiation and progression of infectious diseases, and their transient early surge significantly correlates with SARS-CoV-2 viral load in mild patients [33,34,35]. In particular, IP-10 is secreted in response to interferon-gamma (IFNγ) by different cell types including monocytes, endothelial cells, and fibroblasts [36], and it evokes a range of inflammatory responses, acting as a chemotactic agent for dendritic cells, NK cells, monocytes/macrophages, and T cells [37].

Accumulating evidences indicate for IP-10 an association with the severity of the disease, making it a useful biomarker for predicting COVID-19 progression [38,39]. Moreover, our group previously demonstrated that IP-10 measured at hospital admission in a cohort of SARS-CoV-2-positive patients positively correlated with disease severity and adverse prognosis and inversely with faster recovery [40]. Furthermore, OPN is involved in stimulation of smooth muscle proliferation of arterioles after endothelial damage, which are events well documented in acute COVID-19 physiopathology [41,42] but that may be also associated with the development of a fibrotic phenotype, so OPN could represent a potential predictor factor of pulmonary fibrosis that may occur in post-COVID syndrome [17,18,19,25,43,44]. Further investigations are needed to confirm this hypothesis. from a pathophysiologic point of view, it is not surprising that, according to our report, high OPN concentration in patients with severe prognosis is associated with the increased inflammatory status responsible for the irreversible or hardly reversible respiratory failure. Our data confirm and expand in a larger and more homogeneous cohort of patients the observation reported by other authors [45,46]. Hayek and colleagues demonstrated that elevated circulating levels of OPN in 341 patients treated for COVID-19 in four tertiary care centers in four Western countries directly reflect disease severity and represent an independent risk factor for a more severe clinical course [47]. We described that plasma OPN levels measured at hospital admission above a cut-off of 437 ng/mL predict with good accuracy a worse prognosis. All these finding, taken as a whole, indicate that OPN might contribute to accurate early diagnosis and prognosis for pauci-symptomatic patients that access COVID-19 hospital wards. The plasma level assessment of this protein has the potentiality to be integrated in a panel of known biomarkers that have been described so far for a reliable risk stratification of the patients [40,48,49,50,51]. It is noteworthy that none of the biomarkers described so far in the literature are specific for this disease, thus indicating that integration would be the optimal approach.

This study has several limitations since it focuses on patients hospitalized for COVID-19 with moderate or severe symptoms. Thus, it is not possible to extend our results directly to patients with mild symptoms or even to asymptomatic patients. Furthermore, the single-center nature of this study and a multi-center prospective endorsement of the results obtained is mandatory before recommending the measurement of OPN in clinical practice. Despite these limitations, OPN assessment may enrich the diagnostic tools to help stratify COVID-19 patients’ severity at admission to hospital wards and to plan more specific therapies at the very early stage of the disease.

## 5. Conclusions

This study is a prospective observational cohort that evidenced the possibility of using the plasma OPN concentration at hospital admission to predict which patients might undergo a more severe COVID-19 evolution. Therefore, baseline OPN levels determined at admission to hospital wards might represent a promising biomarker for early stratification of patients’ COVID-19severity. Taken as a whole, our results highlight the role of OPN in COVID-19 evolution, particularly in dysregulated immune-response conditions.

## Figures and Tables

**Table 1:** Demographic characteristics and hospital admission (baseline, t0) clinical conditions of the patients included in this study. IQR, interquartile range. ^§^ refers to data obtained with oxygen supplementation.

Demographics, Parameters, and Clinical Scores	Median (IQR)
Gender	81 males (60.9%)–52 females (39.1%)
Age (years)	63.8 (56.0–72.0)
Heart rate (beats/min)	85 (75–95)
Respiratory rate (breaths/min) ^§^	21 (18–26)
SpO_2_ (%) ^§^	96 (94–98)
Temperature (°C)	36.5 (36.1–36.7)
Systolic pressure (mm Hg)	129 (120–140)
Diastolic pressure (mm Hg)	75 (70–85)
NEWS2	5 (4–6)
Days from illness onset to hospital admission	6 (4–8)
Hemoglobin (g/dL)	14.2 (12.6–15.1)
RDW-CV (%)	13.4 (12.9–14)
White blood cells (cell count × 10^3^/µL)	7.39 (5.09–9.52)
Neutrophils (cell count × 10^3^/µL)	5.7 (5.09–9.52)
Lymphocytes (cell count × 10^3^/µL)	0.71 (0.54–0.97)
Platelets (cell count × 10^3^/µL)	205 (162–256)
ALT (U/L)	37 (28–55)
AST (U/L)	42 (32–57)
Bilirubin (mg/dL)	0.6 (0.5–0.8)
Creatinine (mg/dL)	0.81 (0.64–0.96)
Glomerular filtration rate (mL/min)	89 (70–103)
CRP (mg/dL)	8.26 (4.4–12.97)
LDH (U/L)	718 (550–887)
Erythrocyte sedimentation rate (mm/h)	40 (26–54)
Troponin I (ng/mL)	7 (3–15)
Ferritin (ng/mL)	838.5 (413.0–1354)
D-dimer (µg/L)	721 (517–1328)
Albumin (g/dL)	4.0 (3.7–4.2)
IL-6 (pg/mL)	11.5 (5–31.5)
pO_2_ (mm Hg)	70.0 (59.5–80)
pH	7.46 (7.44–7.49)
pCO_2_ (mm Hg)	37 (31.1–39.0)
PiO_2_/FiO_2_	146 (119.2–176.67)

**Table 2:** Negative disease evolution. Patients’ follow-up and OPN levels correlation. The levels of OPN were assayed with ELISA at day 0 (baseline, t0) and at day 7 (t7) of hospitalization. The table resumes the correlation data between patients with negative disease evolution (in-hospital death or ICU admission) vs. all other patients. Values are expressed as median IQR. N, number of analyzed patients. Bold text evidence statistically significant results.

	Negative Disease Evolution (ng/mL)	All Other Patients (ng/mL)	Z	*p*-Value
t0	(N = 29) 563 (439–720)	(N = 104) 424 (328–587)	2.4494	0.0143
t7	(N = 12) 568 (402–732)	(N = 70) 410 (284–649)	1.2398	0.2151

**Table 3:** Faster clinical recovery. Patients’ follow-up and OPN levels correlation. The levels of OPN were assayed with ELISA at day 0 (baseline, t0) and at day 7 (t7) of hospitalization. The table resumes the correlation data between patients with a faster clinical recovery ((NEWS2) ≤ 2 for at least 24 h in the first 2 weeks or discharge) vs. all other patients. Values are expressed as median (IQR). N, number of analyzed patients. Bold text evidence statistically significant results.

	Faster Clinical Recovery (ng/mL)	All Other Patients (ng/mL)	Z	*p*-Value
t0	(N = 85) 427 (339–563)	(N = 48) 527 (324–733)	−1.6233	0.1045
t7	(N = 53) 400 (275–649)	(N = 29) 498 (363–699)	−0.8050	0.4208

**Table 4:** Logistic regression multivariate. We performed a logistic regression multivariate stepwise to examine the correlation between OPN levels at baseline (t0) and negative disease evolution measured as in-hospital death or ICU admission after demographic and clinical severity variables correction. NEWS2 score and PiO2/FiO2 did not enter in the model.

Predictors	Coefficient	Standard Error	*p*-Value	Odds Ratio	95% Confidence Interval
OPN ng/mL	0.0019	0.0009	0.0422	1.0100	1.0010–1.0138
Age	0.0788	0.0232	0.0007	1.0819	1.0339–1.1322
Sex (female)	−1.7330	0.5782	0.0027	0.1768	0.0569–0.5490

**Table 5:** Multiple correlation analyses between laboratory parameters related to clinical severity or inflammation status and OPN levels (ng/mL) measured at hospital admission (baseline t0). Statistically significant results are evidenced in bold text.

Laboratory Parameters (Determined as)	Correlation Coefficient (OPN vs. Lab. Parameter)	*p*-Value	R^2^
Hemoglobin (g/dL)	−0.1032	0.237	0.011
RDW-CV (%)	0.1624	0.062	0.026
White blood cells (cell count × 10^3^/µL)	−0.1620	0.062	0.026
Neutrophils (cell count × 10^3^/µL)	−0.1457	0.094	0.021
Eosinophils (cell count × 10^3^/µL)	0.0535	0.540	0.003
Lymphocytes (cell count × 10^3^/µL)	0.0008	0.992	0.000
Platelets (cell count × 10^3^/µL)	−0.1485	0.088	0.022
ALT (U/L)	−0.1145	0.193	0.013
AST (U/L)	−0.0626	0.482	0.004
Bilirubin (mg/dL)	0.0115	0.898	0.000
Creatinine (mg/dL)	−0.0641	0.464	0.004
Glomerular filtration rate (mL/min)	−0.0409	0.640	0.002
**CRP (mg/dL)**	**0.1728**	**0.047**	**0.030**
LDH (U/L)	0.129	0.885	0.000
Erythrocyte sedimentation rate (mm/h)	−0.0204	0.835	0.000
Troponin I (ng/mL)	−0.1365	0.123	0.019
Ferritin (ng/mL)	0.0678	0.451	0.005
D-dimer (µg/L)	0.0808	0.366	0.007
Albumin (g/dL)	0.0638	0.480	0.004
**IP-10 (pg/mL)**	**0.1703**	**0.050**	**0.029**
Eotaxin (pg/mL)	−0.0653	0.455	0.004
FGF (pg/mL)	−0.0635	0.468	0.004
G-CSF (pg/mL)	−0.0380	0.664	0.001
GM-CSF (pg/mL)	−0.0542	0.536	0.003
IFN-γ (pg/mL)	−0.1133	0.194	0.013
IL−1 (pg/mL)	−0.0556	0.525	0.003
IL-1 Ra (pg/mL)	−0.0771	0.378	0.006
IL-2 (pg/mL)	−0.0493	0.573	0.002
IL-4 (pg/mL)	−0.0533	0.543	0.003
IL-5 (pg/mL)	−0.0869	0.320	0.008
IL-6 (pg/mL)	−0.0329	0.707	0.001
IL-7 (pg/mL)	−0.0397	0.650	0.002
IL-8 (pg/mL)	0.0154	0.861	0.000
IL-9 (pg/mL)	−0.0137	0.876	0.000
IL-10 (pg/mL)	−0.0410	0.639	0.002
IL-12 (pg/mL)	−0.0720	0.410	0.005
IL-13 (pg/mL)	−0.0783	0.370	0.006
IL-15 (pg/mL)	−0.0405	0.644	0.002
IL-17 (pg/mL)	−0.0550	0.530	0.003
**MCP-1 (pg/mL)**	**0.1856**	**0.032**	**0.034**
MIP-1α (pg/mL)	−0.0186	0.832	0.000
MIP-1β (pg/mL)	−0.0003	0.997	0.000
PDGF (pg/mL)	0.0498	0.569	0.002
RANTES (pg/mL)	−0.1265	0.147	0.016
TNF-α (pg/mL)	−0.0599	0.493	0.004
VEGF (pg/mL)	0.1308	0.1332	0.017
Fibrinogen	−0.3314	0.056	0.110

## Data Availability

The original contributions presented in the study are included in the article. Further inquiries can be directed to the Pier Paolo Sainaghi.
